# PI3K/AKT/mTOR axis in vascular malformations: from molecular insights to targeted clinical trials

**DOI:** 10.1186/s13023-025-04115-2

**Published:** 2025-12-22

**Authors:** Yuan-Yang Zheng, Chen Hua, Xiao-Xi Lin

**Affiliations:** Department of Plastic & Reconstructive Surgery, Shanghai Ninth People’s Hospital, Shanghai Ninth People’s Hospital, Affiliated to Shanghai Jiao Tong University School of Medicine, Shanghai, 200011 China

**Keywords:** Vascular malformations, PIK3CA-related overgrowth spectrum, PI3K/aKT/mTOR pathway, Targeted therapy, Sirolimus, Alpelisib

## Abstract

**Background:**

Vascular malformations are congenital disorders characterized by abnormal blood and/or lymphatic vessels, often leading to pain, functional impairment, and severe complications. Recent advances in molecular genetics have identified key mutations and signaling pathway aberrations, particularly in the PI3K/AKT/mTOR axis, as critical contributors to the etiology of vascular malformations.

**Main body:**

The PI3K/AKT/mTOR axis regulates essential endothelial cell processes, including proliferation, migration, and metabolism. Aberrant activation of this pathway is strongly linked to slow-flow vascular malformations and PIK3CA-related overgrowth spectrum, and increasing evidence also implicates it in fast-flow vascular malformations. These findings underscore the pathway as a promising therapeutic target. This review summarizes current mechanistic insights into PI3K/AKT/mTOR signaling in vascular malformations and examines the therapeutic potential of targeted inhibitors. By integrating results from clinical trials with emerging molecular research, it aims to guide clinical practice while providing future directions for translational investigation.

**Conclusion:**

Recognition of PI3K/AKT/mTOR dysregulation provides a rationale for pathway-directed treatment. Development of selective agents and rational combination strategies—guided by molecular profiling and validated in preclinical and clinical studies—will be essential to enhance efficacy while minimizing resistance and toxicity.

## Background

Vascular malformations are a group of congenital disorders characterized by networks of abnormal blood and/or lymphatic vessels with weak endothelial cell (EC) proliferation. They can occur anywhere in the body and have a significant impact on patients’ life quality. Common symptoms include pain, deformation, esthetic issues, and functional impairment, often leading to complications such as bleeding, recurrent infections, thrombosis and even death [1].

The diagnosis of vascular malformations present a significant challenge due to the complexity and variability in their presentation and clinical course [2, 3]. Treatment strategies are equally diverse, ranging from observation to interventional procedures and surgery, tailored to the specific type of anomaly and its associated complications [4]. However, there is a need for therapeutic options, especially for large and extensive vascular malformations, which cannot be managed with either interventional radiologic techniques or surgical resection [5].

Recent advances in molecular genetics have revealed crucial genetic mutations and signaling pathway aberrations associated with these conditions [6]. Many pathways are implicated, including the angiopoietin/TIE2, PI3K/AKT/mTOR, EPHB4/Ras/MEK/ERK, as well as G protein–coupled receptor signaling molecules such as GNAQ, GNA11, and GNA14 [7]. Angiopoietin/TIE2 mutations, predominantly observed in venous malformations, activate downstream PI3K signaling, which in turn can amplify TIE2 activity through reinforcing feedback loops [8]. Mutations in EPHB4/RAS and GNAQ/GNA11/GNA14 drive MAPK/ERK activation in fast-flow and capillary malformations, respectively, and these pathways exhibit extensive crosstalk with PI3K/AKT/mTOR pathway. Research has consistently shown that overactivation of PI3K/AKT/mTOR pathway is associated with PIK3CA-related overgrowth spectrum (PROS) and slow-flow vascular malformations. Interestingly, cumulative evidence suggests that an excessive activation of this pathway also contributes to arteriovenous malformations (AVM) formation [9, 10]. Thus, despite the heterogeneity of genetic alterations across vascular malformations, PI3K/AKT/mTOR pathway consistently emerges as the central hub, governing EC growth, migration, cytoskeletal remodeling, and metabolism [11]. Given its critical role, this review focuses on the PI3K/AKT/mTOR axis in vascular malformations, examining its mechanisms and highlighting potential targeted therapies for complex or refractory cases.

## PI3K/AKT/mTOR signaling pathway

**Fig. 1:**
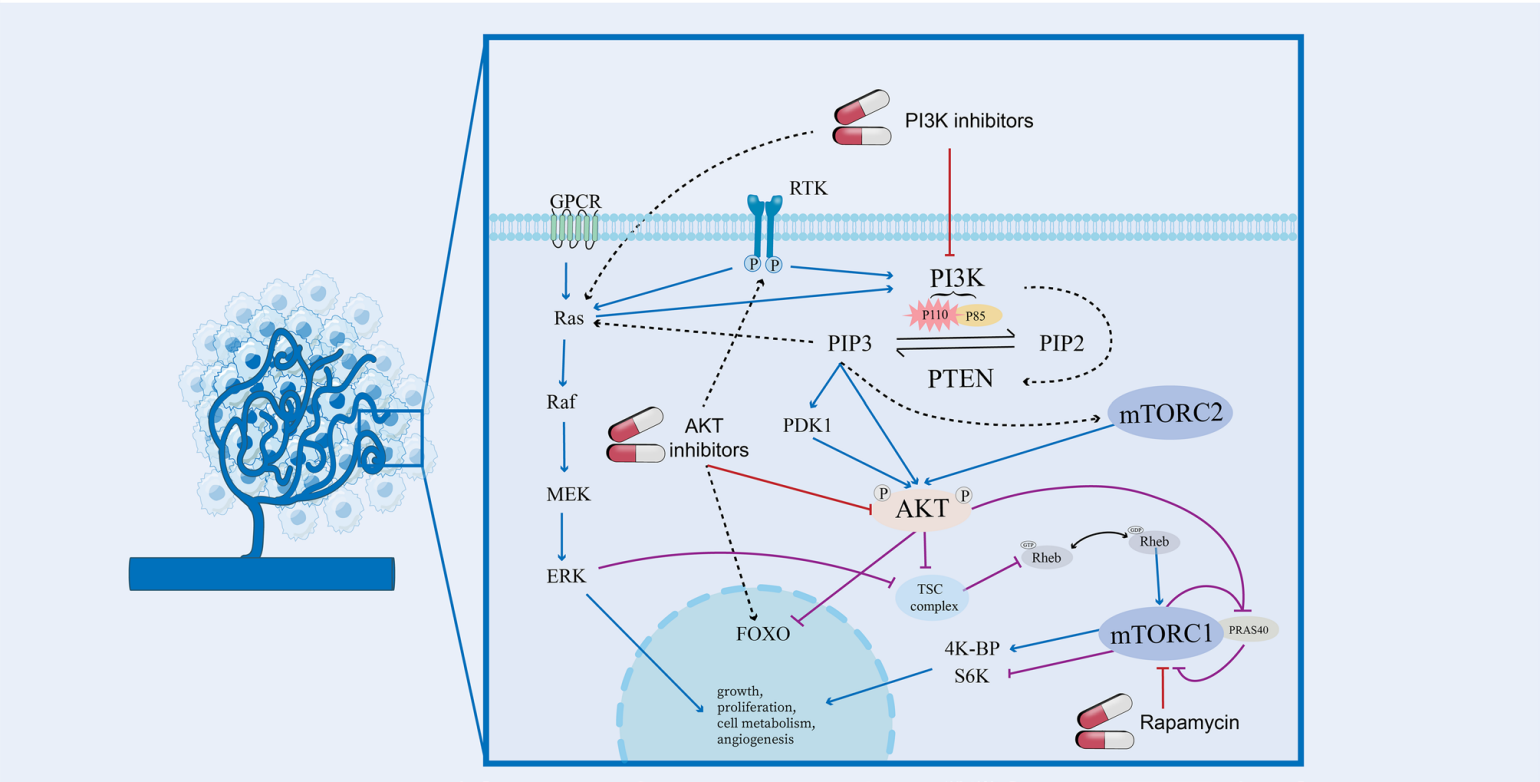
PI3K/AKT/mTOR signaling pathway in vascular malformation this illustration depicts the PI3K pathway, highlighting its activation and regulation, crosstalk with Ras/MEK/ERK axis, and the impact of inhibitors on the pathway

The PI3K/AKT/mTOR pathway is a critical cellular signaling network that regulates essential functions such as cell metabolism, growth, proliferation, survival, and angiogenesis [12–15]. Highly conserved in eukaryotic cells, this pathway responds to external stimuli like growth factors, transmitting signals from cell surface receptors to the nucleus [16]. It interacts extensively with other cellular networks, highlighting its central role in cell biology [17]. Under physiological conditions, it is activated when a ligand binds to its receptor tyrosine kinase (e.g. IGF to IGFR or angiopoietin to TIE2), leading to phosphorylation of phosphatidylinositol-4,5-bisphosphate (PIP2) to phosphatidylinositol-3,4,5-trisphospate (PIP3) via the PI3K family, subsequently recruiting and regulating its downstream target AKT. PTEN serves as a negative regulator of the PI3K/AKT/mTOR pathway by counteracting PI3K activity and thereby inhibiting AKT activation (Fig. 1).

### PI3K

PI3Ks, constitute a family of enzymes that catalyze the phosphorylation of phosphatidylinositides at the 3’ position of the inositol ring [18]. They are divided into three classes, on the basis of protein structure and lipid substrate preference [19].

Class IA PI3Ks, of specific interest in the context of vascular malformations are heterodimers consisting of catalytic subunits (p110α, p110β, and p110δ, encoded by PIK3CA, PIK3CB, and PIK3CD, respectively) in complex with a p85-type regulatory subunit (of which there are five isoforms, encoded by PIK3R1, PIK3R2, and PIK3R3) (Fig. 1) [20]. The regulatory subunits stabilize the overall conformation and inhibit kinase activity in the basal state. On the other hand, direct binding between the BH domain of p85α and PTEN stabilizes PTEN by protecting it from WWP2-mediated proteasomal degradation. This interaction also triggers PTEN lipid phosphatase activity and membrane association, thereby enhancing PTEN activity and downregulating PI3K signaling (Fig. 1) [21, 22]. Thus, the regulatory subunits exhibit a dual role in the PI3K pathway; they can either have a positive (via p110) or a negative (via PTEN) role. Recent study suggests that the monomer-dimer equilibrium of free p85 acts as gatekeeper for PI3K pathway activation [23].

Upon receptor tyrosine kinase activation, the p85/p110 complex is recruited to phosphotyrosine residues on the activated receptor via p85 (SH2 domains) and is preferentially localized to focal adhesions (FAs) for activation [24]. This relieves the p85-mediated inhibition of the p110 catalytic subunit and brings the complex into proximity with its lipid substrates, enabling the phosphorylation of PIP2 into PIP3. PIP3 localizes in a previously-unappreciated ring-shaped sub-domain at the cilia transition zone, distal to PIP2. PI3K signaling network is proven to regulate primary cilia disassembly, and ciliopathy phenotypes can be induced by constitutive in vivo activation of PI3Kα [25]. PIP3 serves as a major second messenger molecule, recruiting effectors which then activate signaling cascades [26, 27]. Recent evidence also highlights the role of PI3K as a signaling hub in mechanosensing and early biomechanical signal transmission in response to tension, stretching, and compression of the plasma membrane, as well as shear stress [28]. Through PI3K signals, every type of mechanical stress will notably impact membrane tension or the cytoskeleton [29, 30].

### AKT

AKT, also known as protein kinase B (PKB), is a 57-kDa serine/threonine kinase. There are three isoforms of AKT in the mammalian genome: AKT1, AKT2, AKT3, among which AKT1 is primarily involved in cellular survival and growth and is critical for VEGF-induced angiogenesis. Recently, lymphatic endothelial cell (LEC) specific CXCR4 deficient embryos exhibited severe defects in lymphatic sprouting, migration, and lymphatic valve formation, revealing a novel mechanism by which CXCR4 modulates VEGFR3/AKT signaling [31]. Akt family proteins consist of several distinct domains: a central kinase domain specific for serine or threonine residues of substrate proteins, an amino terminus pleckstrin homology (PH) domain that mediates lipid-protein and protein-protein interactions, and a carboxyl terminus hydrophobic and proline-rich domain [32].

For the activation of AKT, the translocation of AKT to the inner surface of the plasma membrane by binding to PIP3 through its PH domain is of vital importance. Since PIP3 is generated and enriched around FAs, AKT1, as its effector protein, is dynamically recruited to these FAs for activation. This spatial recruitment/activation of the PI3K-PIP3-AKT cascade is regulated by activated FA kinase (FAK) [24]. The conformational change of AKT resulted from the binding makes it more accessible to PDK1-mediated phosphorylation of Thr308 (Fig. 1). Activated AKT is capable of phosphorylating a wide range of downstream effectors including mTOR and other proteins central to the regulation of apoptosis, transcription factors, and oncogenic factors [33].

### mTOR

**Fig. 2:**
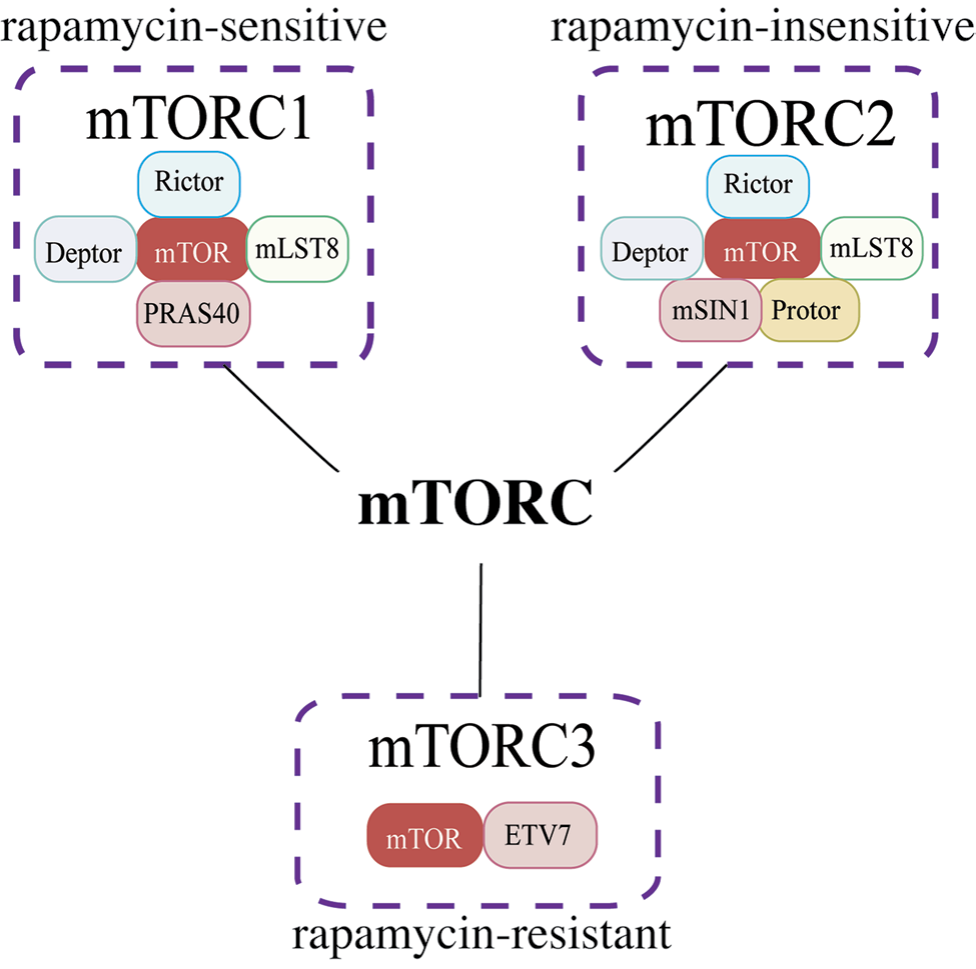
Different subunits of mTORC

The mammalian Target of Rapamycin (also known as mechanistic target of rapamycin, mTOR) is a highly conserved dual-specificity protein kinase that phosphorylates serine/threonine and tyrosine residues [34, 35]. The mTOR has been considered a member of the PI3K family due to the structural similarity between its catalytic domain and that of lipid kinases like PI3K [36]. It is a core component of multiple complexes, notably mTORC1, mTORC2, and a putative rapamycin-resistant mTORC3 [37](Fig. 2).

mTORC1 and mTORC2 form the foundation of a highly integrated signaling network with AKT. AKT activates mTORC1 by phosphorylating and inhibiting the TSC1/2 complex, which acts as a negative regulator of mTORC1 [38]. mTORC1 regulates anabolic processes and cellular growth through key downstream effectors like ribosomal protein S6 kinase (S6K) and 4E-binding protein (4E-BP), which control mRNA translation and protein production [39, 40]. mTORC2, by comparison, phosphorylates AKT at Ser473, facilitating its full activation and enhancing AKTs function [41, 42]. The feedback loop between mTORC2 and AKT also amplifies mTORC1 activity, further strengthening the interconnectedness of the signaling pathway. This system collectively regulates energy production, protein synthesis, autophagy, and cell cycle in G1/S and G2/M phase (Fig. 1) [43, 44].

mTORC3, a recently identified complex, differs from the canonical mTORC1 and mTORC2 complexes as it lacks the Raptor, Rictor, and mLST8 mTORC1/2 components (Fig. 2) [37]. mTORC3 phosphorylates mTORC1 and mTORC2 targets and contains the ETS transcription factor ETV7, which binds to mTOR and is essential for mTORC3 assembly in the cytoplasm. Zhan J et al. also demonstrated that mTOR FRB and LBE sequences in the kinase domain interact with the pointed (PNT) and ETS domains of ETV7, respectively, and that forced expression of the mTOR FRB domain in the mTORC3-expressing, rapamycin-resistant cell line Karpas-299 out-competes mTOR for ETV7 binding and restores rapamycin sensitivity in vivo [45].

### PTEN

PTEN, is encoded by a tumor suppressor gene located on chromosome 10q23. The protein is composed of 403 amino acids and has a dual-specificity phosphatase activity. It includes a phosphatase domain, which plays a key role in regulating PI3K/AKT/mTOR signaling pathway by dephosphorylating PIP3 back to PIP2, and a C2 domain, which binds phospholipid membrane and regulates its activity and localization (Fig. 1). Dimeric p85α has been reported to bind PTEN, positively regulating PTEN membrane association, stability and phosphatase activity [46]. Loss or mutation of PTEN results in overactivation of PI3K pathway, contributing to unchecked cellular processes.

The regulation of PTEN is highly complex and continuously evolving as new mechanisms are discovered. PTEN is controlled at multiple levels, including transcriptional regulation, microRNA-mediated post-transcriptional control, and post-translational modifications such as phosphorylation, ubiquitination, and oxidation [47]. These modifications influence PTENs stability, localization, and activity, with emerging studies constantly revealing additional regulatory layers that impact its tumor suppressor functions and metabolic roles. Furthermore, PTEN is intricately linked with other signaling pathways. YAP/TAZ-EZH2-MYC transcriptional regulators form a nuclear complex that represses PTEN transcription, while their combinatorial targeting restores PTEN expression [48]. Accumulated and activated PTEN leads to inactivation of Akt/mTORC and promotion of auto-lysosomal degradation of YAP1 involving p62/Sqstm1 and lysosomal cathepsins (CtsH and CtsL) [49]. CD44/Brg1/PRMT5 regulatory module functions to repress PTEN [50]. Spatial analysis reveals a miRNA-related PTEN depletion is strongly associated with severe hypoxia and increased cell proliferation [51].

### Pathway feedback

PI3K pathway is modulated by negative feedback, where activation of downstream signaling leads to inhibition of upstream signaling, including multiple receptor tyrosine kinases (RTKs). PI3K-AKT inhibitors relieve this feedback and reactivate RTK signaling which may attenuate their antitumor activity. This is partly due to the crosstalk between pathways: AKT inhibition triggers FOXO-dependent RTK expression [52], while PI3K inhibition activates the MAPK pathway via SH2 domains [53]. mTORC1 also initiates powerful negative feedback regulation of growth factor receptor signaling, such that inhibition of mTORC1 or S6K1 leads to elevated activation of PI3K, AKT, and the ERK pathway (Fig. 1) [36, 54]. These insights offer new strategies to address drug resistance in targeted therapies [55].

A novel mechanism has been proposed in which PI3K regulates PTEN translation via mTOR/4E-BP1, thus buffering changes in PI3K signaling [56]. Physiologic or oncogenic activation of PI3K/AKT/mTOR signaling leads to increased PTEN expression that limits the duration of the signal and prevents overactivation. Conversely, inhibition of the pathway reduces PTEN levels, blunting inhibitory effects. It provides an explanation for the co-occurrence of PTEN loss and PI3K mutation in some cancers and offers new insights into overcoming resistance to PI3K inhibitors.

## Roles of PI3K/AKT/mTOR axis in vascular malformations

Overactivation of PI3K/AKT/mTOR signaling pathway is central to the etiology of many vascular malformations, as this pathway represents a major hub that determines EC fate including EC growth, migration, actin cytoskeleton remodeling, metabolism, and vesicular trafficking. This overactivation can result from excessive physiological stimulation by upstream regulators or gain-of-function mutations in genes involved in the PI3K/AKT/mTOR pathway, including PIK3CA, PIK3R1, PIK3R2 and AKT1 [11, 57, 58]. They are specifically associated with PROS and vascular malformations in vessels characterized by slow fluid flow, including venous, lymphatic and capillary malformations (Table 1). On contrary, these mutations have not been reported in arterial malformations according to human spectrum of vascular malformations [59, 60]. The basis of such vessel type–restricted disease manifestation remains obscure. Speculations on why arteries remain unaffected by these mutations suggest that it may be due to the refractory behavior arterial cells (ECs) exhibit upon reaching their definitive maturation state [61, 62], along with cell cycle arrest induced by fluid shear stress on arterial ECs [63].

However, cumulative evidence suggests that an excessive activation of PI3K pathway contributes to AVM formation. Elevated PI3K/AKT signaling has been identified in both HHT patient skin biopsies and mutant ECs in HHT mouse models, and inhibiting this pathway successfully rescues AVMs in mice [9, 10]. This implies that fast and slow flow vascular lesions might share common molecular features and further proved the importance of excessive PI3K/AKT/mTOR activation in the etiology of vascular malformations.

Activating mutations in PIK3CA are predominantly somatic, as their germline mutations disrupt vasculogenesis/angiogenesis and caused lethality during embryonic development [64, 65].

Hot-spot mutations in PIK3CA include p.Glu542Lys, p.Glu545Lys (helical domain), and p.His1047Arg (kinase domain) [66, 67]. Interestingly, despite the fact that these same PIK3CA mutations are one of the most common oncogenic mutations in cancer [67]^,^ vascular malformation associated with PIK3CA mutation rarely acquire a malignant phenotype. An important difference is that vascular malformation is characterized by a single copy of the mutant PIK3CA (heterozygous) which most likely arise in progenitor cells during development. On contrary, cancer-causing mutations arise in differentiated cells, leading to multiple oncogenic hits along the same pathway, which results in susceptibility to additional oncogenic mutations and a stem-like phenotype [68, 69].

**Table 1:** Mutated genes associated with PI3K axis identified in vascular malformations

Mutated genes	Malformations	Reference
PIK3CA	Lymphatic Malformation (LM)	Osborn et al. [70], Luks et al. [71]
	Venous Malformation (VM)	Limaye et al. [66]
	PROS	
	Capillary lymphatic venous malformations (CLVM)	Le Cras et al. [68]
	Klippel-Trénaunay syndrome (KTS)	Luks et al. [71]
	CLAPO syndrome	Rodriguez-Laguna et al. [72]
	CLOVES syndromes	Kurek et al. [73]
	Facial infiltrating lipomatosis (FIL)	Maclellan et al. [74]
	Megalencephaly-capillary malformation-polymicrogyria (MCAP)	Park et al. [75]
PIK3R1/PIK3CA	Capillary Malformation with Dilated Veins (CMDV)	De Bortoli et al. [76]
PIK3R1	PIK3R1-related vascular overgrowth syndrome	Kuentz et al. [58]
AKT1	Proteus syndrome	Lindhurst et al. [77]
PTEN	PTEN hamartoma tumor syndrome	Tan et al. [78]

### Venous malformations and lymphatic malformations

Venous malformations (VMs) are, with the estimated incidence of 1/5000, the most frequent vascular malformation treated in specialized centers [1]. They present as soft, compressible subcutaneous masses with bluish skin discoloration, and are usually devoid of bruit, pulsation, or redness. Lymphatic malformations (LMs) are characterized by dilated lymphatic channels or cysts lined with LECs, often disconnected from the normal lymphatic system [79]. In LMs, dilated channels are filled with lymphatic and blood fluids [80], suggesting there could be an incomplete separation from the blood circulation [81].

PIK3CA mutations are found in approximately 80% LM cases [71]. In-vitro studies using human LM-derived ECs have demonstrated that PIK3CA-mutant LECs exhibit PI3K/AKT overactivation, leading to increased proliferation and sprouting properties. These pro-angiogenic activities can be suppressed with treatment of either PI3K or AKT inhibitors [81]. A new immune-interacting subtype of Ptx3-positive dermal lymphatic capillary endothelial cells (iLECs) is proven to recruit pro-lymphangiogenic macrophages to promote progressive lymphatic overgrowth in Pik3ca^H1047R^ mice [82].

Approximately 25–30% of VMs are caused by somatic gain-of-function mutations in the PIK3CA gene, which encodes the p110α isoform of phosphoinositide 3-kinase (PI3K) [83]. In addition, activating somatic mutations were identified in almost half of sporadic VM cases in the gene coding for the endothelial-specific tyrosine kinase receptor TEK (TIE2), which result in enhanced activation of downstream PI3K/AKT pathways [84]. Correlation between different types of mutations and phenotypes reveals that, in contrast to TEK-mutant VMs, PIK3CA-mutant VMs present more lymphocytic aggregates, deeper lesions not extending into the skin, and lower phosphorylated AKT (p-AKT) levels [66, 85]. Research into the pathogenic mechanisms of these mutations is actively ongoing. PIK3CA mutations, when expressed in human umbilical vein endothelial cells (HUVECs), caused chronic activation of AKT, disrupted normal EC-characteristic monolayer morphology, resulted in loss of ECM fibronectin and strongly dysregulated certain important angiogenic factors [66].

In fact, since lymphatic vessels stem from primitive lymph sacs within veins [86], LMs and VMs might derive from the same cell lineage at different embryonic stages. For example, PIK3CA mutation should be acquired before lymphatic specification occurs during vascular development in capillary lymphatic venous malformations (CLVM), based on research on EC populations [68]. In-vitro studies have demonstrated that PIK3CA-mutant ECs generate pathological proliferative response only upon stimulation of growth signals [60, 68, 87], which corresponds with clinical observations where low-flow vascular malformations arise during embryonic development, expand proportionally with the patient’s physiological growth and remain largely quiescent during adulthood, when the production of growth factors is minimal. However, acute production of external stimuli, such as hormonal changes and inflammatory processes, can reactivate proliferation in the malformed vasculature [82, 88]. Study of loss-of-function genetic models has unraveled that subunit p110α plays a critical role in regulating collective cell migration during angiogenesis [89]. Meanwhile, defective cell migration can cause capillary-venous malformations when ECs are unable to re-distribute within the vascular network [90]. It is tempting to speculate that aberrant EC migration also accounts for the pathogenic mechanism induced by activating mutations in PIK3CA in lymphatic, venous malformations.

### PROS

PROS are a group of diseases with heterogeneous segmental overgrowth phenotypes caused by somatic activating mutations of PIK3CA. Many of them are associated with vascular malformations, such as Klippel-Trénaunay syndrome (KTS), CLOVES syndrome, diffuse capillary malformation with overgrowth (DCMO) and megalencephaly-capillary malformation-polymicrogyria (MCAP) [91, 92]. More recently, it has been proposed to broaden PROS to ‘PI3K-related overgrowth spectrum’ to include phenotypes linked to both PIK3CA and PIK3R1 variants, as mutations in PIK3R1, encoding the p85-α, p55-α or p50-α regulatory subunit of PI3-kinases, have also been found in patients with PROS-like phenotypes [58].

They are associated as hyperactivation of the PI3K/AKT/mTOR signaling. However, the reason for the significant variation in clinical features among PROS remains uncertain. Whether they depend on the positions of the mutated nucleotide in PIK3CA, the timing of mutations, or cell types in which mutations occur still requires clarification [93].

### AVM

AVMs are fast-flow vascular lesions characterized by abnormal connections between arteries and veins, bypassing the normal capillary bed. Associated with high morbidity and mortality, they are considered the most dangerous and difficult-to-treat vascular malformations [1].

The pathogenesis of AVM remains elusive, while several signaling pathways have been implicated in its development, including MAPK/ERK, Notch, BMP and PI3K [94–96]. Hereditary hemorrhagic telangiectasia (HHT) is an inherited disorder of vascular malformations characterized by mucocutaneous telangiectases and AVMs in internal organs [97]. HHT is classified into three subtypes based on specific genetic mutations: ENG, ACVRL1 (also known as ALK1), and SMAD4. It is frequently used in research to investigate the mechanisms underlying AVM formation. Recently, cumulative evidence indicates that excessive activation of PI3K pathway contributes to AVM formation. Skin biopsies of HHT2 patients showed an increase in the PI3K/AKT pathway [9]. Studies using ALK1 transgenic mouse models of HHT discovered that although an increase in PI3K signaling by itself is not sufficient to trigger AVMs, overactivation of PI3K/AKT pathway was involved in HHT2 mice [98]. Further, inhibition of excessive PI3K or mTOR activity rescued excessive EC proliferation, leading to suppressed or improved AVM formation, which could serve as a putative novel therapeutic approach for HHT2 patients [10, 99, 100].

In vitro studies have shown that BMP9/ALK1 signaling increase PTEN activity via SMAD4 in ECs, thereby decreasing PI3K/AKT signaling [9, 98]. Moreover, SMAD4 limits PI3K pathway activation by regulating the fluid shear stress (FSS) set point. KLF4 overexpression after Smad4 loss mediates excessive PI3K pathway activation in ECs through transcriptional regulation of TIE2. Further, loss of physiological-FSS-induced cell cycle arrest in G1 due to excessive KLF4-mediated repression of CDK inhibitors and resultant loss of arterial identity is a key driver of AVM formation upon SMAD4 depletion [101].

### PTEN hamartoma tumor syndrome

PTEN hamartoma tumor syndrome (PHTS) refers to a spectrum of disorders associated with germline mutations in the PTEN gene, including Cowden syndrome, Bannayan-Riley-Ruvalcaba syndrome, adult Lhermitte–Duclos disease and autism spectrum disorders associated with macrocephaly [102]. In PHTS families, Loss-of-function PTEN mutations are inherited in an autosomal-dominant manner [103–105]. Clinical features of PHTS present a broad variability and there has not been a clear genotype/phenotype correlation shown with PTEN. Because PTEN functions as a negative regulator of PI3K, its loss of function results in hyperactivation of PI3K/AKT/mTOR pathway, leading to overgrowth of a number of tissues (skin, colon, thyroid, etc.). In addition, as PTEN is an important tumor suppressor gene, PHTS patients are predisposed to several types of cancers. Other clinical features include macrocephaly, hamartomatous intestinal polyps, lipomas, developmental delay and vascular anomalies [106]. Recent study showed that lymphoblastoid cell lines (LCLs) from PHTS individuals with autism spectrum disorder (ASD) and/or developmental delay (DD) showed faster DNA damage repairing rate than those from patients without ASD/DD or cancer [107].

## Targeted therapies for vascular malformations

In general, treatment of vascular malformations is primarily surgical, composed of excision and debulking along with interventional therapies such as pulsed laser dye and sclerotherapy [5]. However, these approaches have significant limitations due to the extensiveness of malformations, high relapse rates, and substantial surgical morbidity [6]. Consequently, there is a pressing need for targeted therapies, particularly for large and extensive vascular malformations that cannot be effectively managed through traditional treatment. The advances in understanding the role of PI3K/AKT/mTOR axis in these malformations have opened the era of exploiting targeted therapies, as summarized in Table 2, which highlights current and upcoming clinical trials on PI3K axis inhibitors for vascular malformations.

**Table 2:** Current and upcoming clinical trials on PI3K axis inhibitors for vascular malformations

Drug		Clinical trials	Phase	Trial ID/ Status	Age
mTOR inhibitor	Sirolimus	Sirolimus for Complicated Vascular Anomalies	NA^a^	NCT03583307 Completed	0–18 Years
		Topical Sirolimus for Complicated Vascular Anomalies	I	NCT04172922 Suspended	36 Months to 21 Years
		Sirolimus for LMs	NA	NCT06160739 Recruiting	6 Months to 12 Years
		Sirolimus for Superficial Voluminous Complicated Slow-flow Vascular Malformations (PERFORMUS-II)	II	NCT02509468 Completed	6 Years to 18 Years
		Sirolimus for Vascular Anomalies (VASE-III)	III	NCT02638389 Recruiting	3 Months to 70 Years
		Sirolimus for Cystic LMs	II/ III	NCT06673290 Recruiting	1 Year to 18 Years
		Percutaneous Sirolimus for Superficial Complicated Vascular Anomalies	IV	NCT04921722 Recruiting	0 Years to 18 Years
		Weekly Sirolimus Therapy for VMs and LMs	II	NCT04861064 Recruiting	2 Years and older
		Topical Sirolimus for Microcystic LMs (PALV-06)	II	NCT05050149 Completed	6 Years and older
			III	NCT06239480 Recruiting	
		Rapamycin for Cervico-facial LMs	II	NCT03243019 Unknown	0 Years to 18 Years
		Topical Sirolimus for Cutaneous Microcystic LMs	II	NCT03972592 Unknown	6 Years and older
		Sirolimus for Nosebleeds in HHT	II	NCT05269849 Completed	18 Years and older
	Tacrolimus	Tacrolimus for HHT	II	NCT04646356 Completed	18 Years and older
	CERC-006	CERC-006 for Complex LMs	I	NCT04994002 Withdrawn	18 Years to 31 Years
PI3K inhibitor	Alpelisib	Retrospective Review Study of Alpelisib for PROS (EPIK-P1)	NA	NCT04285723 Completed	2 Years and older
		Alpelisib for LMs Associated with a PIK3CA Mutation (EPIK-L1)	II/ III	NCT05948943 Recruiting	2 Years and older
		Alpelisib for PROS (EPIK-P2)	II	NCT04589650 Active, not recruiting	0 Days to 100 Years
		Long-term Safety and Efficacy of Alpelisib for PROS (EPIK-P3)	II	NCT04980833 Active, not recruiting	2 Years and older
		Alpelisib for MCAP (SESAM)	II	NCT05577754 Recruiting	2 Years to 40 Years
		Targeted Therapies for Slow-Flow or Fast-Flow Vascular Malformations (TARGET-VM)	II	NCT05983159 Recruiting	2 Years and older
	CYH33	CYH33 for PROS and PRVM^b^	II	CTR20231410 Recruiting	18 Years and older
	VT30	Topical VT30 for Venous, Lymphatic or Mixed Malformations	I/ II	NCT04409145 Part1 completed, Part 2 terminated	18 Years to 60 Years
AKT inhibitor	VAD044	VAD044 for HHT	I/ II	NCT05406362 Active, not recruiting	18 Years and older
	Miransertib	Miransertib for Proteus Syndrome	I	NCT02594215 Completed	6 Years to 65 Years
		Miransertib for PROS and Proteus Syndrome	I/ II	NCT03094832 Terminated	2 Years and older
		Long-term safety of Miransertib for PROS and Proteus Syndrome	II	NCT04980872 Active, not recruiting	2 Years to 120 Years

^a^Non Applicable

^b^PIK3CA-Related Vascular Malformation

### mTOR inhibitors

Sirolimus (Rapamune or rapamycin) is a macrocyclic lactone produced by the bacteria Streptomyces hygroscopicus, and prevents phosphorylation of proliferation and survival-related proteins by inhibiting the mTOR complex 1. Discovered in the early 1970s, it is currently approved by the US Food and Drug Administration (FDA) as an immunosuppressant to prevent kidney allograft rejection in children ≥13 years old and used as immunosuppressive, antiangiogenic, and cytostatic agent in clinical practice [108, 109].

Sirolimus exhibits efficacy and safety for slow-flow vascular malformations from infancy to adults [110–113]. Dosing varied between studies, but most used 0.8 mg/m^2^ per dose twice daily for children, and 2 mg/day for adults to maintain a serum trough level of 10–15 ng/ml.

The Vascular Anomaly-Sirolimus-Europe (VASE) trial (EudraCT 2015-001703-32, NCT02638389), initiated in 2016, is so far the largest multicentric prospective phase III trial regarding sirolimus in slow-flow vascular malformations [113]. One hundred thirty-two patients (31 patients < 19 years and 101 adult patients) were included, among whom 107 completed 12 or more months of sirolimus, including 61 who were treated for the whole 2-year period. 85% of patients had a clinical improvement on sirolimus within the first year, and sirolimus increased feasibility of surgery or sclerotherapy in 20 (15%) patients initially deemed unsuitable for intervention. Among the 61 patients who completed the 2-year treatment, 33 (54%) reported a recurrence of symptoms after a median follow-up of 13 months after sirolimus arrest. Clinical improvement was faster but subsided more rapidly in PIK3CA-mutated (*n* = 24) compared with TIE2-mutated (*n* = 19) patient, although there was no difference in efficacy.

A prospective, multicenter phase II trial in China (NCT03583307), which included 126 pediatric patients with complicated VAs, showed that sirolimus was able to deliver outstanding responses, not only in patients with VM, common LM, but also in those with KHE and combined malformations with a prominent venous and/or lymphatic component. In contrast, response rates to sirolimus were lower for those with AVMs [111]. Another clinical trial PERFORMUS (NCT02509468) demonstrated that children with pure LM had the best radiographic and clinical response compared to other slow-flow malformation types [112]. Meanwhile, in a prospective study on 39 patients of PROS, it’s suggested that low-dose sirolimus (target serum level of 2–6 ng/ml) can modestly reduce overgrowth, but cautions that the side-effect profile is significant, mandating individualized risk-benefit evaluations for sirolimus treatment in PROS. Medical therapy for PROS is likely to be suppressive rather than curative, and may be indefinite from childhood [114].

While sirolimus has proven to be efficacious in most slow-flow vascular malformations, its long-term safety still remains a major concern, especially for young patients. In the VASE trial, adverse events (AEs) occurred in 96% of patients, with most being mild and manageable, such as mucositis, diarrhea, and rash, while 5% experienced serious AEs. Although a systematic review suggested that sirolimus is effective and well tolerated within the first year of life, children responded more often and earlier than adults, with lower pain scores, supporting the initiation of sirolimus at an early age [115, 116]. However, until larger-scale clinical trials are conducted, the safety of sirolimus in infants will continue to be approached conservatively.

Efforts to minimize the adverse effects of sirolimus in the treatment of vascular malformations have led to two promising strategies: lowering the drug dose and altering the route of administration. In a case series of 12 patients with therapy-resistant low-flow vascular malformations treated with sirolimus at low target levels (4–10 ng/mL), response rates were comparable to those reported with higher sirolimus levels, but with a lower incidence of serious adverse events [117]. Building on this, the ongoing clinical trial NCT06673290 is recruiting patients to assess the outcomes of maintaining sirolimus trough levels between 5 and 8 ng/mL over a 1-year period. Another trial, NCT04861064, aims to reduce dosing frequency by administering sirolimus once weekly (1.5–2 mg/m^2^) for 6 months. Switching from systemic to localized administration is another innovative approach under investigation. The use of topical sirolimus has so far been reported only in scattered case reports [118–120], for instance, in a case series involving 11 patients with superficial LM, topical application of sirolimus (0.4–1%) modified the clinical appearance and alleviated symptoms of superficial LM. As expected, no significant clinical improvement was observed in the deeper lymphatic components of the lesions treated [121]. Currently, several clinical trials are evaluating the efficacy of topical sirolimus formulations for treating superficial vascular anomalies, including capillary microcystic lymphatic malformations (CMLM) and microcystic LMs. These trials involve sirolimus concentrations ranging from 0.1% to 3.9%, offering a localized therapeutic option with potentially reduced systemic side effects (NCT04172922, NCT04921722, NCT03972592, NCT06239480).

Everolimus is a sirolimus derivative, with an additional hydroxyethyl group in its structure. With a shorter half-life, it is absorbed quickly and metabolized faster. Although everolimus showed capacity to treat PIK3CA-mutated mouse model of vascular malformations [122], no further systematic clinical studies were conducted afterward, except several case reports. In clinical management for vascular anomalies, everolimus was much less used than sirolimus [123].

### PI3K inhibitors

#### Pan-PI3K inhibitors

Pan-PI3K inhibitors act by targeting all four isoforms of class I PI3Ks (α, β, γ, and δ), leading to comprehensive inhibition of the PI3K pathway. While these inhibitors have shown some efficacy in various solid tumors, their non-specific targeting often results in significant toxicities, such as liver enzyme elevations, hyperglycemia, and skin reactions [124, 125]. Clinical trials have demonstrated only modest benefits in terms of progression-free survival, particularly in patients with PIK3CA mutations [126–128]. Due to their broad activity and associated adverse effects, pan-PI3K inhibitors have shown limited success across different indications and are thus rarely considered for vascular malformations. This has led to a shift in focus towards more selective inhibitors, aiming to reduce toxicity while preserving therapeutic efficacy.

#### PI3K isoform-specific inhibitors

Alpelisib (BYL719) is a PI3Kα inhibitor originally approved for breast cancer and became the first medical treatment to obtain US Food and Drug Administration approval for PROS in 2022 [129, 130]. In 2018, the first evidence of its efficacy in the first mouse model of PROS/CLOVES that recapitulated the human phenotype was followed by successful clinical application in 19 patients, where alpelisib improved symptoms and organ function with a favorable safety profile, even for patients for whom sirolimus was not an effective option [131]. Shortly thereafter, data from the primary analysis EPIK-P1 (NCT04285723), a non-interventional, retrospective chart review of 57 patients with PROS (≥2 years), was reported and assessed by FDA, which accelerated approval on April 5, 2022, for adult and pediatric patients 2 years of age and older with severe manifestations of PROS who require systemic therapy [132]. Yet, vascular malformations are congenital disorders that commonly manifest with clinical symptoms in the first year of life and early access to treatment is sometimes preferable before the approved age for alpelisib initiation. Off-label therapy in infants has also shown promising efficacy, but it suggests that alpelisib metabolism may be significantly reduced in this population. This necessitates further clinical trials, including pharmacokinetic monitoring, and mandates close and careful monitoring to avoid potential toxicity [133, 134].

So far, Alpelisib has also demonstrated efficacy in-vitro and in-vivo studies in capillary venous malformations [135] and LM [136, 137], and attenuated the proliferation of CLVM EC in a dose-dependent manner. In patients resistant to usual therapies, including sirolimus, debulking surgeries, or percutaneous sclerotherapies, previously intractable vascular malformations were reduced and clinical symptoms were attenuated. Notably, alpelisib halted disease development but did not correct the genetic anomaly, which explains why withdrawal of alpelisib often led to recurrence of tumors. It’s suggested that continuous administration of alpelisib may be needed for sustained control, or this class of drugs can also be envisioned to be utilized in a neoaduvant approach to render large cases resectable [132, 135].

Drug-related adverse events are to be expected during treatment with alpelisib, based on the fact that it is not a mutant-selective PI3Kα inhibitor and that PI3Kα is ubiquitous. Since PI3Kα inhibition interferes with insulin signaling, hyperglycemia was the most frequent adverse effect of alpelisib in treatment of breast cancer [138]. However, in the treatment of vascular malformation, the incidence and intensity of hyperglycemia are relatively lower. This is partly due to the significantly lower doses used compared to cancer treatment and possibly because the patients are younger and have a more robust glucose homeostasis [137]. In the EPIK P1 clinical trial, the most common treatment-related adverse events were hyperglycemia (12.3%), and aphthous ulcers (10.5%) and cellulitis (1.8%). Other typical adverse effects include diarrhea, alopecia and disseminated intravascular coagulation, most of which were within a manageable range and no patients permanently discontinued alpelisib because of an AE.

Although alpelisib appears effective and well tolerated at present, important uncertainties remain regarding the optimal duration of therapy and the potential for resistance. In EPIK-P1, the longest reported treatment reached 49.9 months, with some patients still on therapy, and long-term outcomes are now being evaluated in the Phase II EPIK-P3 study (NCT04980833) [132]. A randomized, placebo-controlled trial (EPIK-P2; NCT04589650) is ongoing in approximately 150 pediatric and adult patients with PROS to further assess efficacy, safety, and pharmacokinetics. Additionally, a multi-center Phase II trial is investigating alpelisib in pediatric and adult patients with Megalencephaly-Capillary Malformation Polymicrogyria Syndrome (MCAP), where the passage of alpelisib through the blood-brain barrier will also be evaluated (NCT05577754) [139].

Apart from alpelisib, other PI3Kα isoform-selective inhibitors are also being explored. Taselisib was assessed for six-month tolerability and efficacy in PROS patients [140]. Despite some functional improvement, the safety profile of low-dose taselisib limits its potential for long-term use. CYH33, another selective PI3Kα inhibitor, is being evaluated in a multicenter phase I/II study to assess its safety, tolerability, pharmacokinetics, and efficacy in patients with PROS and PIK3CA-related vascular malformations (PRVM), with results pending disclosure (CTR20231410). Due to the high prevalence of PI3K mutations in cancer [141], there has been considerably more research on PI3K inhibitors in solid tumors compared to vascular malformations. For example, GDC-0077 (Inavolisib), a highly selective inhibitor and degrader of mutant PI3Kα, has demonstrated efficacy in breast cancer. Early-phase trials have shown a favorable safety profile, and a Phase III clinical trial is currently underway to further evaluate its effectiveness in breast cancer [142, 143]. More clinical studies are needed to explore the use of these inhibitors in treating vascular malformations.

#### PI3Kα mutant-selective inhibitors

To minimize the impact on WT PI3K and reduce adverse effects, new inhibitors specifically targeting p110α variants are being developed to enhance treatment efficacy in mutant cells while improving safety profiles. Among them, RLY-2608, the first known allosteric, pan-mutant, and isoform-selective PI3Kα inhibitor, has proven its potential for improved tolerability and efficacy in both in-vitro and in-vivo studies [144]. Remarkably, it not only demonstrated the ability to overcome resistance mediated by secondary PI3Kα mutations but also minimized the impact on insulin signaling, reducing hyperglycemia and allowing for higher target engagement and a wider therapeutic index [145]. The ongoing phase I/II ReDiscover trial (NCT05216432) will further define the potential benefit of RLY-2608 in patients with advanced PIK3CA-mutant solid tumors and breast cancer. Another pan-mutant PI3Kα inhibitor RLY-5836, molecularly distinct with differentiated pharmaceutical properties, has also initiated its first-in-human study recently (NCT05759949) in patients with advanced solid tumors harboring a PIK3CA mutation [146].

LOXO-783 is an allosteric, brain-penetrant small-molecule inhibitor with high selectivity toward the p.H1047R variant of PI3Kα. Based on its preclinical activity in in-vitro models without causing hyperglycemia, a phase 1 trial (PIKASSO-01) is being conducted in PIK3CA H1047R-mutant advanced breast cancer and other solid tumors [147]. Meanwhile, STX-478 and pyridopyrimidinone compound 17, both structures were recently disclosed, presented high selectivity for mutant PI3Kα and provided tumor regressions and a clear pharmacodynamic response in mice [148, 149].

### AKT inhibitors

Miransertib (ARQ-092, MK-7075), a selective allosteric pan-AKT inhibitor, has shown promise in treatment of Proteus syndrome and PROS. In a phase 0/1 pilot study of miransertib in patients with Proteus syndrome, 5 out of 6 participants achieved the primary endpoint of a 50% reduction in pAKT phosphorylation in affected tissues within four months of treatment initiation [150]. Additionally, a 5-year follow-up of an individual who continued treatment beyond the initial study supports the long-term safety of miransertib [151]. Subsequent anecdotal cases provided evidence that treatment of miransertib led to significant improvement in both benign and malignant conditions, as observed in a patient with Proteus syndrome and a recurrence of low-grade serous ovarian carcinoma [152]. In primary fibroblasts obtained from 6 miransertib-treated PROS patients, miransertib showed higher anti-proliferative activity with lower cytotoxicity compared to mTOR inhibitors [153]. Furthermore, miransertib was administered to two children with PROS, resulting in initial improvements and no significant toxicities; however, treatment was discontinued due to a lack of sustained response and poor compliance [154].

Preclinical models have also provided evidence of miransertib’s potential. It effectively inhibited PI3K/AKT signaling and reduce cell viability in patient-derived PIK3CA- and TEK-mutant ECs [155] and exhibited greater potency than sirolimus in inhibiting cell proliferation in CLVM patients-isolated ECs [68]. In PIK3CA^Tie2R-CreER^ mice, miransertib led to a modest reduction in VM volumes and extended lifespan [135]. Additionally, in a mouse model of PI3K-driven vascular malformation with EC-specific-Pik3ca^H1047R^ expressions, miransertib not only prevented disease progression but also fully regressed established vascular malformations, indicating its promise as a therapeutic strategy for both PIK3CA and TEK-mutant vascular malformations [155].

Despite these promising results, the clinical development of miransertib has faced challenges. The phase I/II clinical trial, designed to assess the safety and tolerability of miransertib in participants aged 2 years and older with PROS or Proteus syndrome, was terminated due to business reasons (NCT03094832). Another highly selective AKT inhibitor, MK2206, which targets all three isoforms of AKT (Akt1, Akt2, and Akt3), has also shown potential in preclinical studies, such as blocking LEC proliferation in vitro [156], a significant decrease in p-AKT and other downstream effects in TIE2-mutated ECs [157].

VAD044, an oral, once-daily allosteric AKT inhibitor, represents a novel therapeutic approach specifically designed for the treatment of hereditary hemorrhagic telangiectasia (HHT). A randomized, placebo-controlled, double-blind, multicenter phase 1b study (NCT05406362) is underway to evaluate the safety and efficacy of VAD044 in adult patients with HHT. Preliminary analysis revealed a reduction in mean epistaxis frequency by approximately 33% among patients treated with 40 mg of VAD044 who completed the study. Recognizing its potential, the FDA has granted Fast Track Designation to VAD044 for the treatment of adults with HHT, highlighting its promise in addressing the unmet medical needs in this population.

### PTEN restoration

PTEN is classified as a tumor suppressor gene, and targeting tumor suppressors poses significant challenges [158]. One major difficulty is that in many cancers, PTEN is completely lost, rendering it impossible to target or reactivate [159, 160]. Even when PTEN is present but mutated, restoring its normal function is inherently complex, as reactivating a malfunctioning tumor suppressor protein involves intricate processes. Additionally, PTEN loss often results from epigenetic or transcriptional silencing, which further complicates direct gene targeting [158, 161–164].

Despite these challenges, alternative therapies aimed at managing PTEN deficiency have been explored in other disease areas, particularly in oncology. Recent advances include gene therapy approaches such as PTEN plasmid delivery and nanocarrier-based systems, which show potential in drug-resistant melanoma [165]. Similarly, PTEN gene nanovectors are being investigated for lung cancer [166], while clinical studies in prostate cancer have demonstrated the ability of PTEN gene therapy to enhance chemotherapy efficacy [167]. Furthermore, mRNA-based therapies are emerging as promising strategies to restore PTEN function and boost antitumor immunity in preclinical models [168].

As PTEN restoration and related therapies continue to evolve, there is growing potential to apply these strategies beyond cancer treatment. Vascular malformations may benefit from targeting the dysregulated PI3K/AKT/mTOR pathway through PTEN restoration or mimetic compounds, potentially helping to modulate aberrant cellular growth and improve patient outcomes.

## Conclusion

With the discovery that somatic mutations activating the PI3K/AKT/mTOR pathway are central to various vascular malformations, and the continuous advancements in genomic sequencing technologies, our understanding of the pathophysiological mechanisms underlying these conditions has deepened substantially. The identification of mutations in key genes that activate critical signaling pathways has provided the foundation for more personalized treatment approaches. Given the commonalities between these pathways and those involved in other diseases, such as cancer, clinical trials have swiftly adapted by repurposing existing drugs, showing promising outcomes.

Looking forward, the development of more precise and selective therapeutic targets will be essential in advancing the treatment of vascular malformations. Multi-target approaches that simultaneously inhibit multiple points within these pathways [169], coupled with combination therapies, hold the potential to enhance treatment efficacy while minimizing resistance. Such strategies could allow for lower doses and reduced side effects, tailored specifically to the genetic makeup of each patient. As we move toward these future therapies, ongoing in vitro and in vivo research, along with carefully designed clinical trials, will be crucial in bringing these innovations from the laboratory to the clinic, ultimately offering improved and more personalized care for patients with vascular malformations.

## Data Availability

Not applicable.

## Abbreviations

4E-BP: 4E-Binding Protein
ALK1: Activin Receptor-Like Kinase 1
ASD: Autism Spectrum Disorder
AVM: Arteriovenous Malformation
BMP: Bone Morphogenetic Protein
CDK: Cyclin-Dependent Kinase
CLAPO: Capillary Malformation of the Lower Lip, Lymphatic Malformation of the Face and Neck, Asymmetry, and Partial/Generalized Overgrowth
CLOVES: Congenital Lipomatous Overgrowth, Vascular Malformations, Epidermal Nevi, and Skeletal/Spinal Anomalies
CLVM: Capillary Lymphatic Venous Malformation
CMDV: Capillary Malformation with Dilated Veins
CXCR4: C-X-C Motif Chemokine Receptor 4
DD: Developmental Delay
EC: Endothelial Cell
ECM: Extracellular Matrix
ENG: Endoglin
EPH: Ephrin type-B receptor 4
ERK: Extracellular Signal-Regulated Kinase
ETV7: ETS Variant Transcription Factor 7
FA: Focal Adhesion
FAK: Focal Adhesion Kinase
FDA: Food and Drug Administration
FIL: Facial Infiltrating Lipomatosis
FSS: Fluid Shear Stress
HHT: Hereditary Hemorrhagic Telangiectasia
HUVEC: Human Umbilical Vein Endothelial Cell
iLEC: Immune-Interacting Lymphatic Endothelial Cell
IGF: Insulin-like Growth Factor
IGFR: Insulin-like Growth Factor Receptor
KLF4: Krüppel-Like Factor 4
KTS: Klippel-Trénaunay Syndrome
LEC: Lymphatic Endothelial Cell
LM: Lymphatic Malformation
MAPK: Mitogen-Activated Protein Kinase
MCAP: Megalencephaly-Capillary Malformation-Polymicrogyria
MEK: MAPK/ERK Kinase
mTOR: Mechanistic Target of Rapamycin
mTORC: mTOR Complex
PHTS: PTEN Hamartoma Tumor Syndrome
PI3K: Phosphoinositide 3-Kinase
PIK3CA: Phosphatidylinositol-4,5-Bisphosphate 3-Kinase Catalytic Subunit Alpha
PIP2: Phosphatidylinositol-4,5-Bisphosphate
PIP3: Phosphatidylinositol-3,4,5-Trisphosphate
PROS: PIK3CA-Related Overgrowth Spectrum
PTEN: Phosphatase and Tensin Homolog
RTK: Receptor Tyrosine Kinase
S6K: Ribosomal Protein S6 Kinase
SH2: Src Homology 2
SMAD4: SMAD Family Member 4
TAZ: Transcriptional Coactivator with PDZ-Binding Motif
TIE2: Tyrosine Kinase with Immunoglobulin-like and EGF-like Domains 2
TSC: Tuberous Sclerosis Complex
VEGF: Vascular Endothelial Growth Factor
VEGFR3: Vascular Endothelial Growth Factor Receptor 3
VM: Venous Malformation
YAP: Yes-Associated Protein
